# Morphological characteristics of different types of distal radius die-punch fractures based on three-column theory

**DOI:** 10.1186/s13018-019-1453-x

**Published:** 2019-11-27

**Authors:** Jinhua Zhou, Wen Tang, Dong Li, Yongwei Wu

**Affiliations:** 1 Department of Orthopaedics, Liyang People’s Hospital, Liyang, Jiangsu China; 2 Department of Radiology, Wuxi Ninth People’s Hospital Affiliated of Soochow University, Wuxi, Jiangsu China; 3 Department of Radiology, Liyang People’s Hospital, No. 70, Jianshe West Road, Liyang, 213000 Jiangsu China; 4 Department of Orthopaedics, Wuxi Ninth People’s Hospital Affiliated of Soochow University, No.999, Liangxi Road, Binhu District, Wuxi, 214061 Jiangsu China

**Keywords:** Distal radius, Die-punch fracture, Three-column theory

## Abstract

**Objective:**

The aim of this study is to investigate the morphological characteristics of distal radius die-punch fracture (DRDPF) with different types, based on the three-column theory.

**Methods:**

The imaging data of 560 patients diagnosed with DRDPF were reviewed and divided into single-column, double-column, or three-column DRDPF according to the three-column theory, and the types, case distribution of DRDPF, and inter- and intra-agreement of classification were further analyzed.

**Results:**

There were 65 cases of single-column DRDPF, 406 cases of double-column DRDPF, and 89 cases of three-column DRDPF. Among the single-column DRDPF, there were three cases of volar, 13 cases of dorsal, 14 cases of split, and 35 cases of collapse type fractures. Among the radius column fracture, there were 130 cases of metaphseal,155 cases of articular surface, and 210 cases of combined type. The inter-observer Kappa coefficient was 0.877–0.937, and the intra-observer kappa was 0.916–0.959, showing high agreement. At the 12th month’s follow-up, according to the Gartland–Werley score system for the functionary recovery of the wrist and hand, 519 cases (92.68%) of the patients ranked excellent or good, and 41 cases (7.32%) ranked fair. All the cases were fair results, and the intermediate column of the distal radius was collapse type fractures, showing significant difference between the collapse type and other types (χ2 = 23.460, *P* = 0.000). The excellent and good rate in the single-, double-, and three-column DRDPFs were 93.85%, 92.16%, and 91.01%, respectively (χ2 = 0.018, *P* = 0.991).

**Conclusion:**

Due to the difference of the nature and energy of the forces, the position of wrist, and the bone quality of the patients at the moment of the injury, the loading forces transmitted to the intermediate column of the distal radius could result in different types of DRDPF. The classification method in this study included all types of DRDPF, indicating the mechanism, affected sites, and the morphological characteristics of DRDPF with high consistency, which hopefully could provide insight into the treatment and prognosis of DRDPF patients.

## Introduction

A die-punch is the articular surface fracture resulting from axial impact and compressive forces [1–3]. In 1962, Scheck initially referred the dorsal articular surface avulsion fracture of the lunate fossa of the distal radius as die-punch fracture fragment. Later, the articular surface fracture of the lunate fossa resulting from the load transmitting through the lunate was referred to as the distal radius die-punch fracture (DRDPF). The most common type was the collapse fracture of the articular surface of the lunate fossa [4–8]. However, the name of die-punch fracture only indicates the mechanism, failing to suggest the fracture sites and bone involvement.

**Fig. 1:**
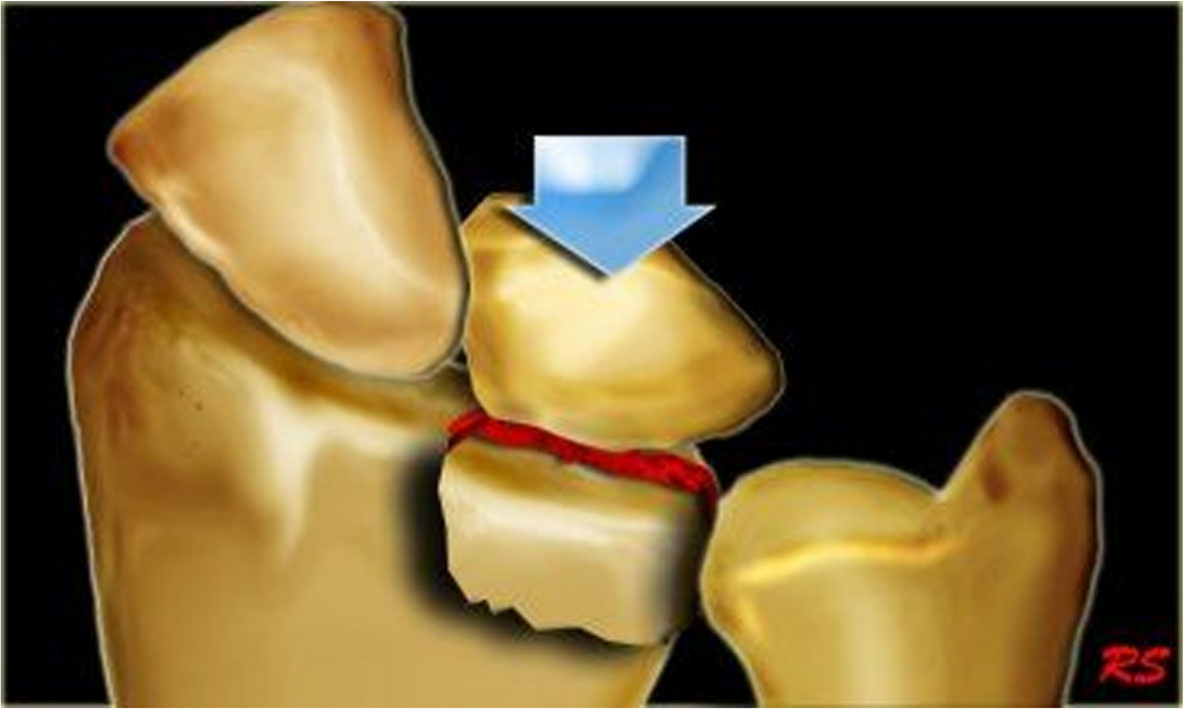
Illustration of distal radius die-punch fracture locates in the ulnar side of the distal radius

**Fig. 2:**
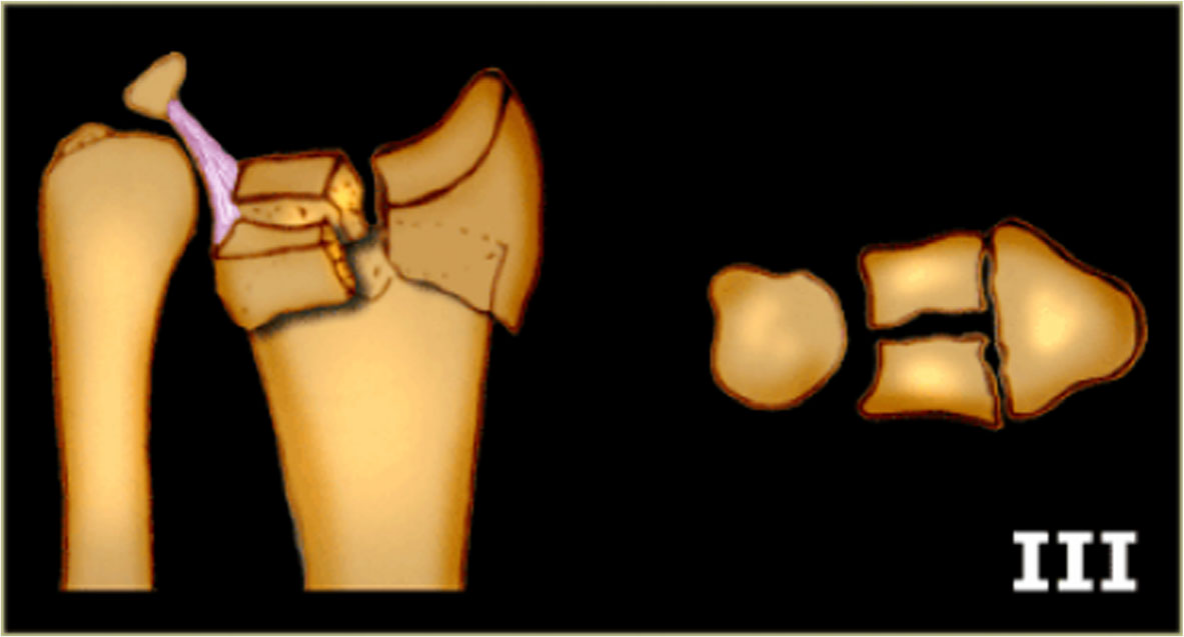
Illustration of distal radius die-punch fracture mainly involves the ulnar demifacet of the radius accompanied by a slight fracture of the radial half of the radius and the styloid process of the ulna). Source: the illustration of type III distal radius fracture by Fernandez classification

**Fig. 3:**
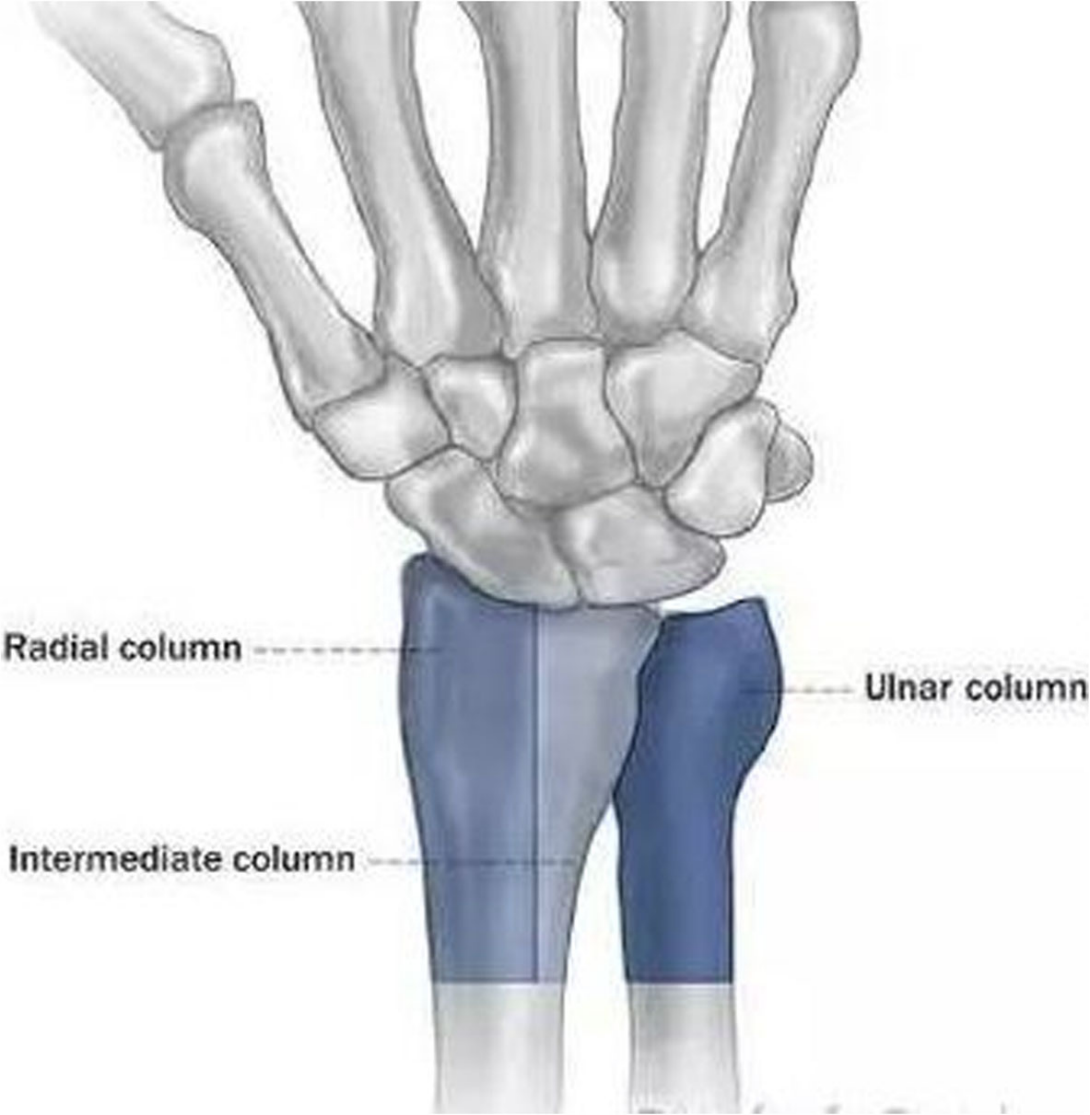
The three-column model of distal radius fracture

Some researchers also included the collapse fracture of other sites resulting from axial loading forces in the list of die-punch fracture fragment or die-punch fracture. For example, the use of distal tibia articular surface die-punch fractures and articular calcaneus die-punch fractures was adopted in previous literature [9, 10]. Due to the difference of the nature and the energy of the forces, the position of wrist, and the bone quality of the patient at the moment of the injury, DRDPF can either involve the ulnar demifacet of the radius (Fig. 1), or mainly involve the ulnar demifacet of the radius in conjunction with mild fracture of the radial half of the radius or and the styloid process of the ulna (Fig. 2). The later one was classified as type II fracture in the Melone classification [11] and type III fracture in the Fernandez classification [12], which is also known as die-punch fracture. According to the “three-column theory” of distal radioulna fracture proposed by Rikl and Regazzoni [13] (Fig. 3), the former should be called intermediate column DRDPF, the latter should be called double- or three-column DRDPF. At present, there are many methods for describing distal radius fractures, among which the AO classification method is widely used, but no studies have focused on the different types of DRDPF. Zhang [14], Ma [10], and Xi [15] have reported the different types of single-column and double-column DRDPF, but there are still a lack of reports on three-column DRDPF and comparative studies on the severity of various DRDPF. Therefore, the existing DRDPF classification system is not sufficient. This study mainly reviewed the clinical data of DRDPF in our hospital and explored the classification and clinical features of various DRDPF.

## Materials and methods

### Inclusion and exclusion criteria

Inclusion criteria: (1) The articular surface fracture of the intermediate column of the distal radius resulting from axial loading forces, or the above being the main fracture in conjunction with mild fracture of the radial column or the styloid process of the ulna. (2) No CT scan request only when the X-ray is very clear. Otherwise, patients must obtain both the X-ray and the CT scans. Exclusion criteria: (1) The open articular surface fracture of the intermediate column of the distal radius resulting from direct violence. (2) The articular surface fracture of the radial column of the distal radius is more prominent than the articular surface fracture of the intermediate column. (3) Patients without complete imaging data.

### General materials

The clinical data of 560 patients was selected based on the above inclusion criteria, including 305 males and 255 females aging from 13 to 89 with an average of 46.41. They were selected among the 23,508 patients who were diagnosed with distal radius fracture with complete imaging data from June 2007 to June 2017 in the Wuxi the Ninth People’s Hospital Affiliated to Soochow University and Liyang People’s Hospital. In terms of the causes, 208 cases occurred due to falling, 149 cases occurred due to tumbling, 122 cases occurred due to traffic accidents, and 81 cases occurred due to direct impact. Some patients suffered combined fractures, among which 59 cases combined with fracture of the styloid process of ulna, 48 cases with distal radiolunar joint laxity or dislocation, and 134 with fractures at other sites. All the patients obtained the AP X-ray; among them, 555 patients (99.11%) underwent CT scans as well.

### Classification methods

Firstly, the single-column, double-column, or three-column DRDPF were distinguished based on the three-column theory and the characteristics of the affected sites. Afterwards, they were further divided into sub-types based on the fracture sites and morphological characteristics with reference to the AO classification.

### The single-column or intermediate-column DRDPF was divided into four sub-types

Single-column or intermediate-column DRDPF was divided into four types according to the methods of Yin [16] and Ma [10]: volar, dorsal, split, and collapse (comminuted fracture and collapse displacement of the central portion of the articular surface of the medial column).

**Fig. 4:**
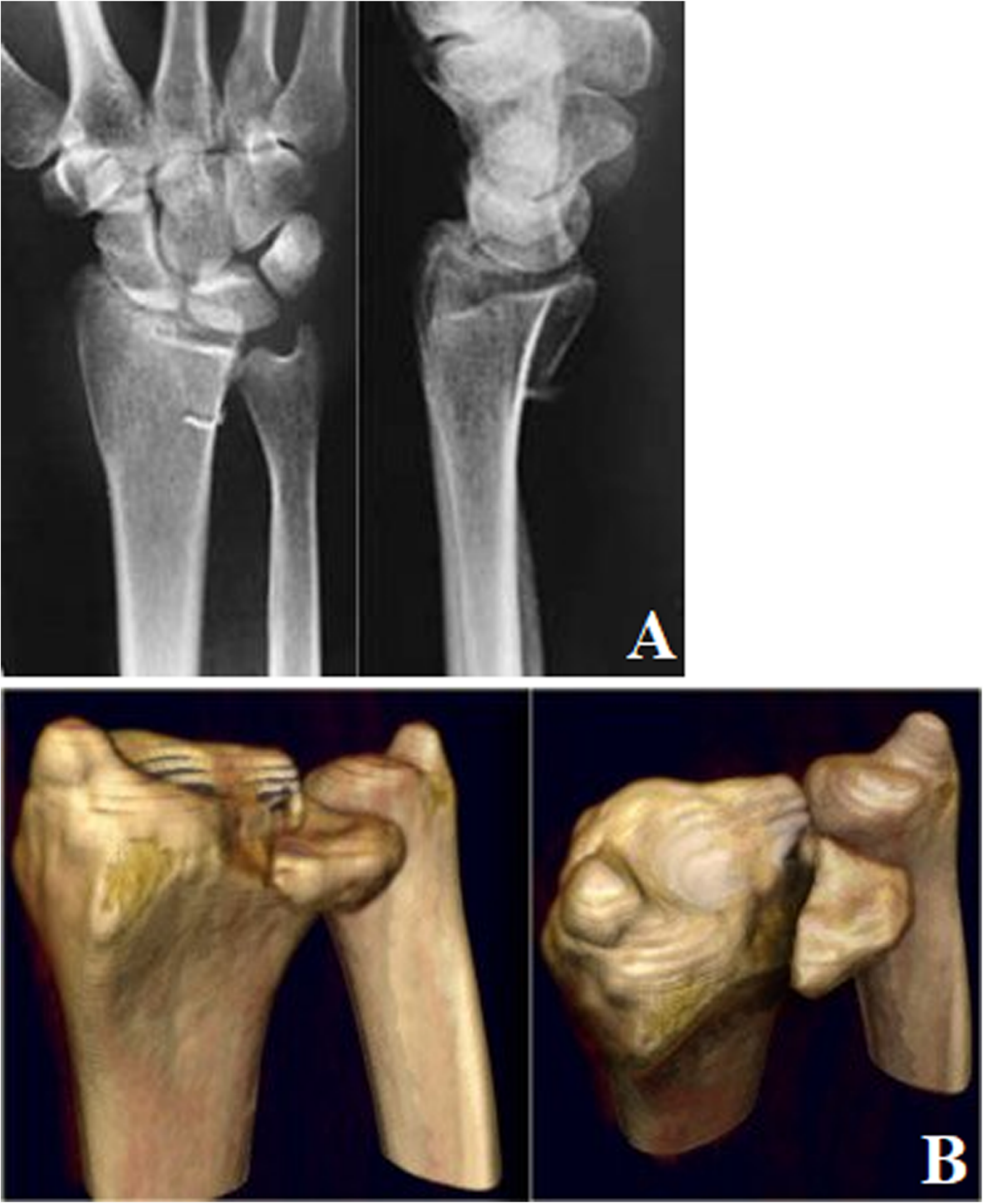
PA X-ray (**a**) and CT scan (**b**) of volar single-column DRDPF

**Fig. 5:**
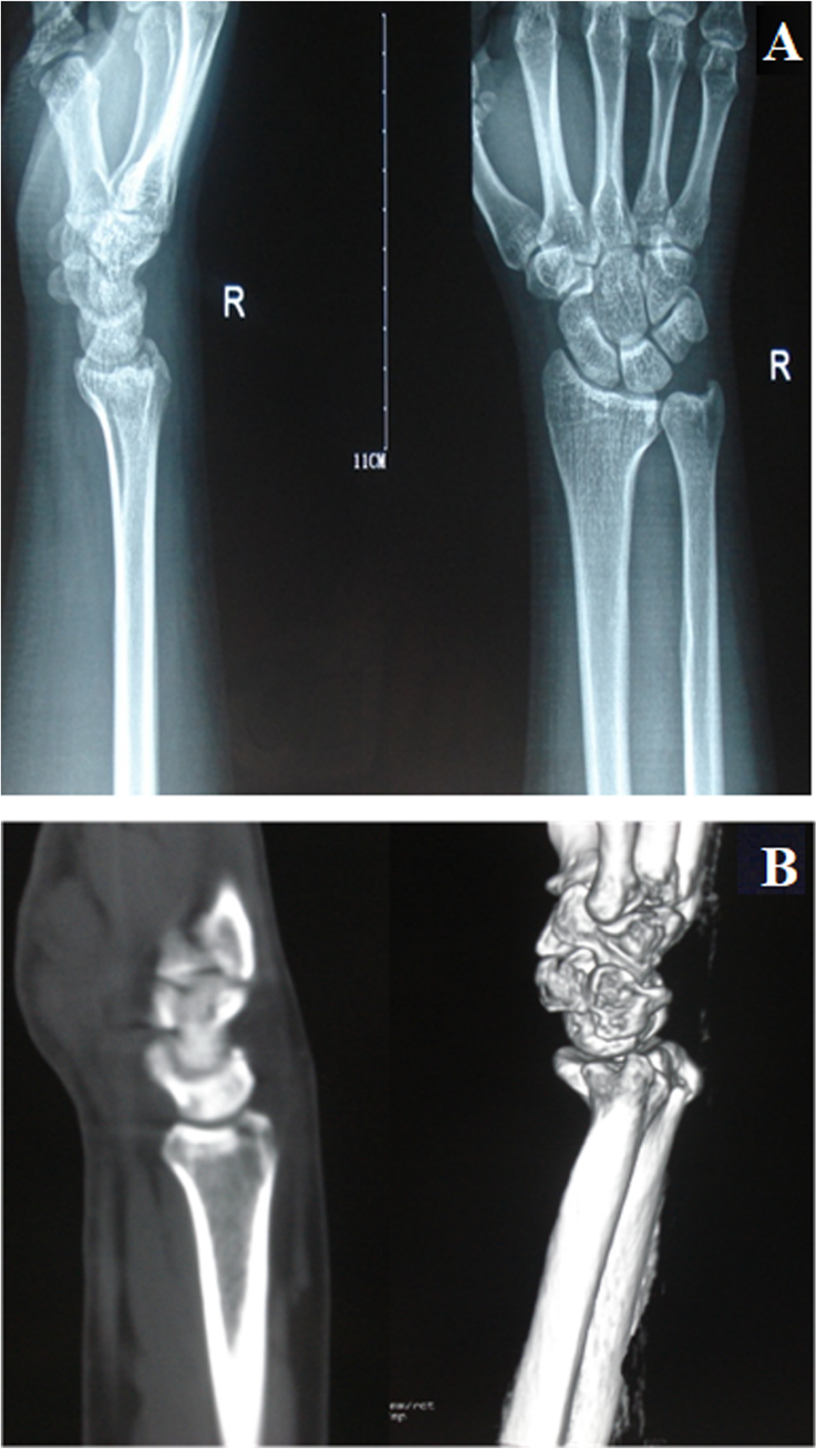
PA X-ray (**a**) and CT scan (**b**) of dorsal single-column DRDPF

**Fig. 6:**
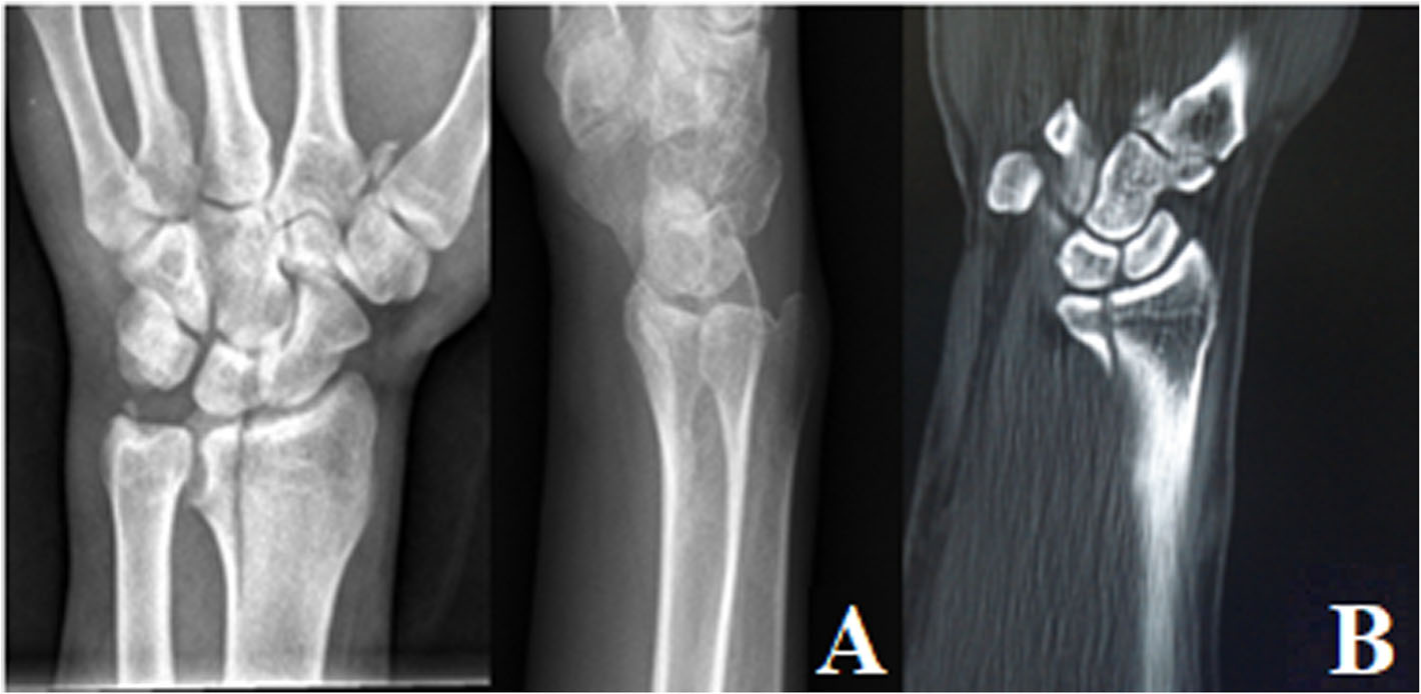
PA X-ray (**a**) and CT scan (**b**) of the spilt single-column DRDPF

**Fig. 7:**
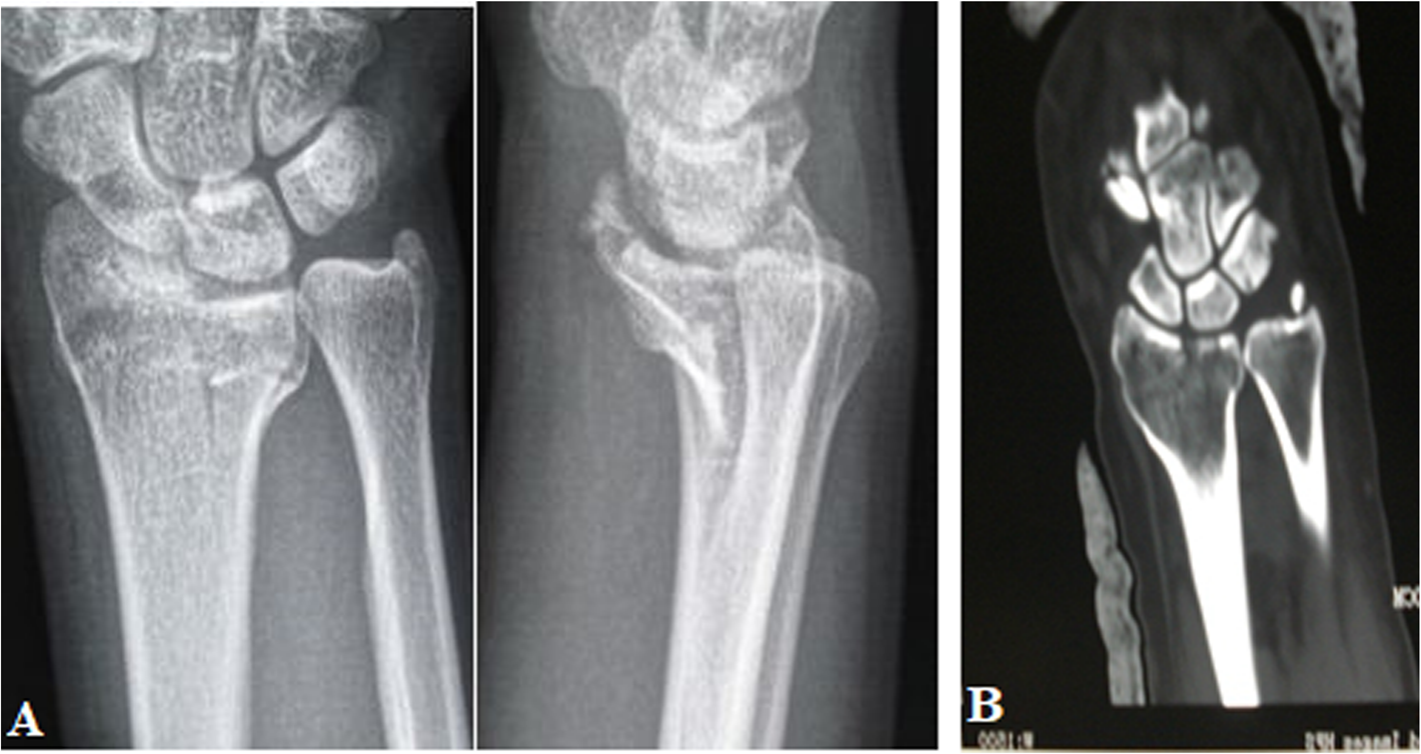
PA X-ray (**a**) and CT scan (**b**) of the collapse single-column DRDPF

1. Volar. The fracture only involved the volar articular surface of the intermediate column of the distal radius. No dorsal fracture was present (Fig. 4).
2. Dorsal. The fracture only involved the dorsal articular surface of the intermediate column of the distal radius. No volar fracture was present (Fig. 5).
3. Split. The longitudinal fracture at the sagittal position of the intermediate column of the distal radius, which was commonly associated with varying degrees of separation. Mostly, no obvious articular comminution or collapse fracture occurred (Fig. 6).
4. Collapse. The articular surface of the intermediate column of the distal radius was centrally, or volarly and dorsally collapsed or comminuted simultaneously. Longitudinal fracture of the sagittal position of the intermediate column and separation could sometimes occur as well (Fig. 7).

### The radius column fracture among the double- or three-column DRDPF was divided into three types

**Fig. 8:**
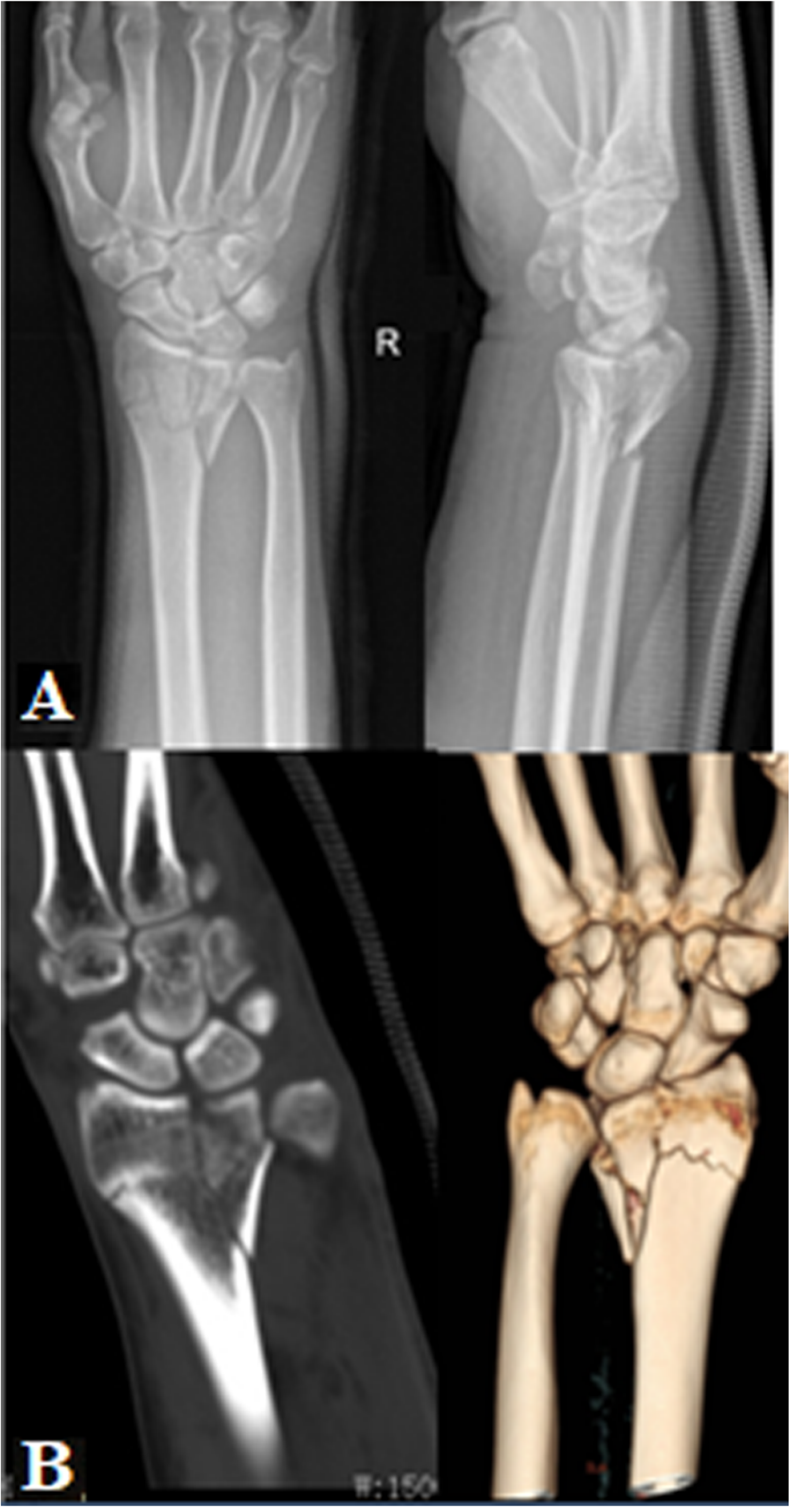
PA X-ray (**a**) and CT scan (**b**) of the metaphyseal radius column fracture

**Fig. 9:**
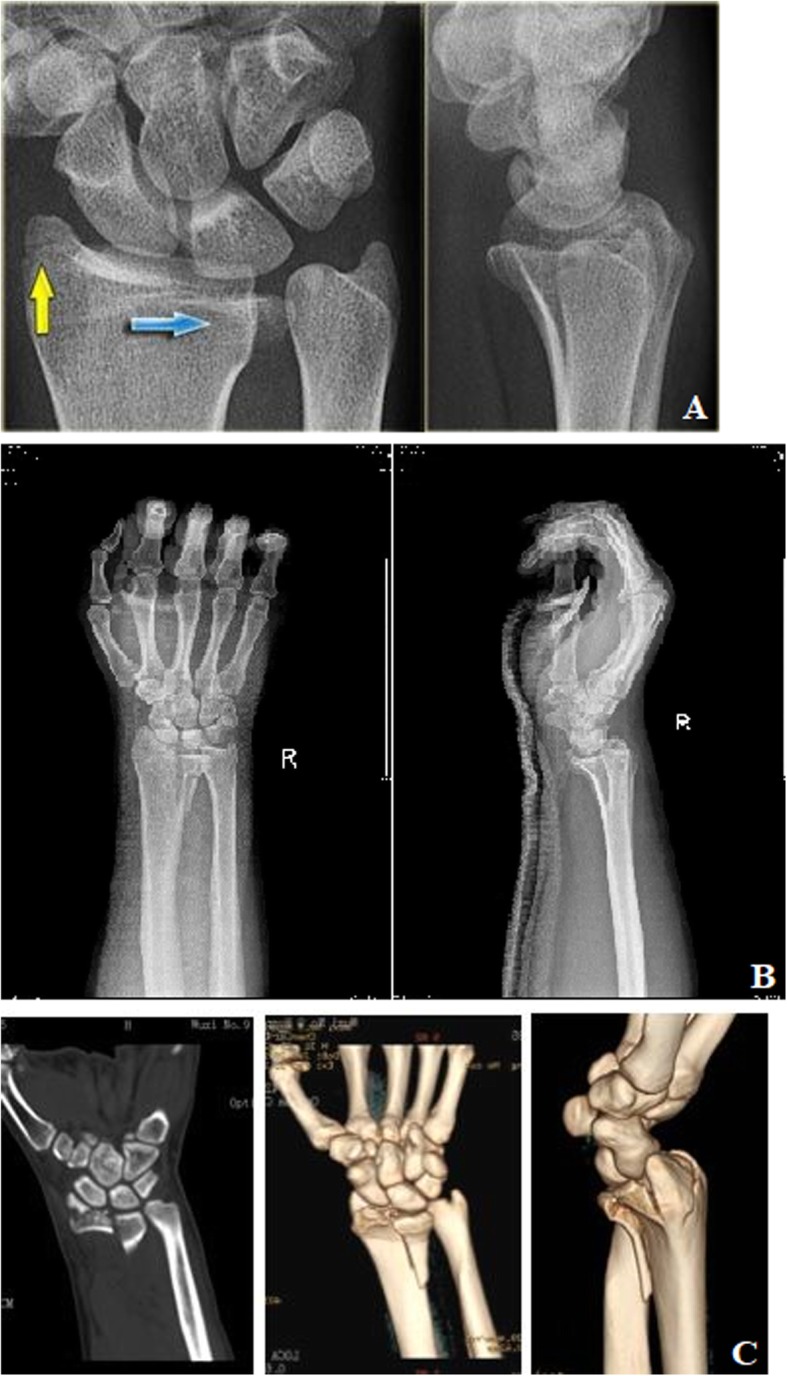
PA X-ray in example 1 (**a**), PA X-ray in example 2 (**b**), and CT scans in example 2 (**c**) of articular surface radius column fracture

**Fig. 10:**
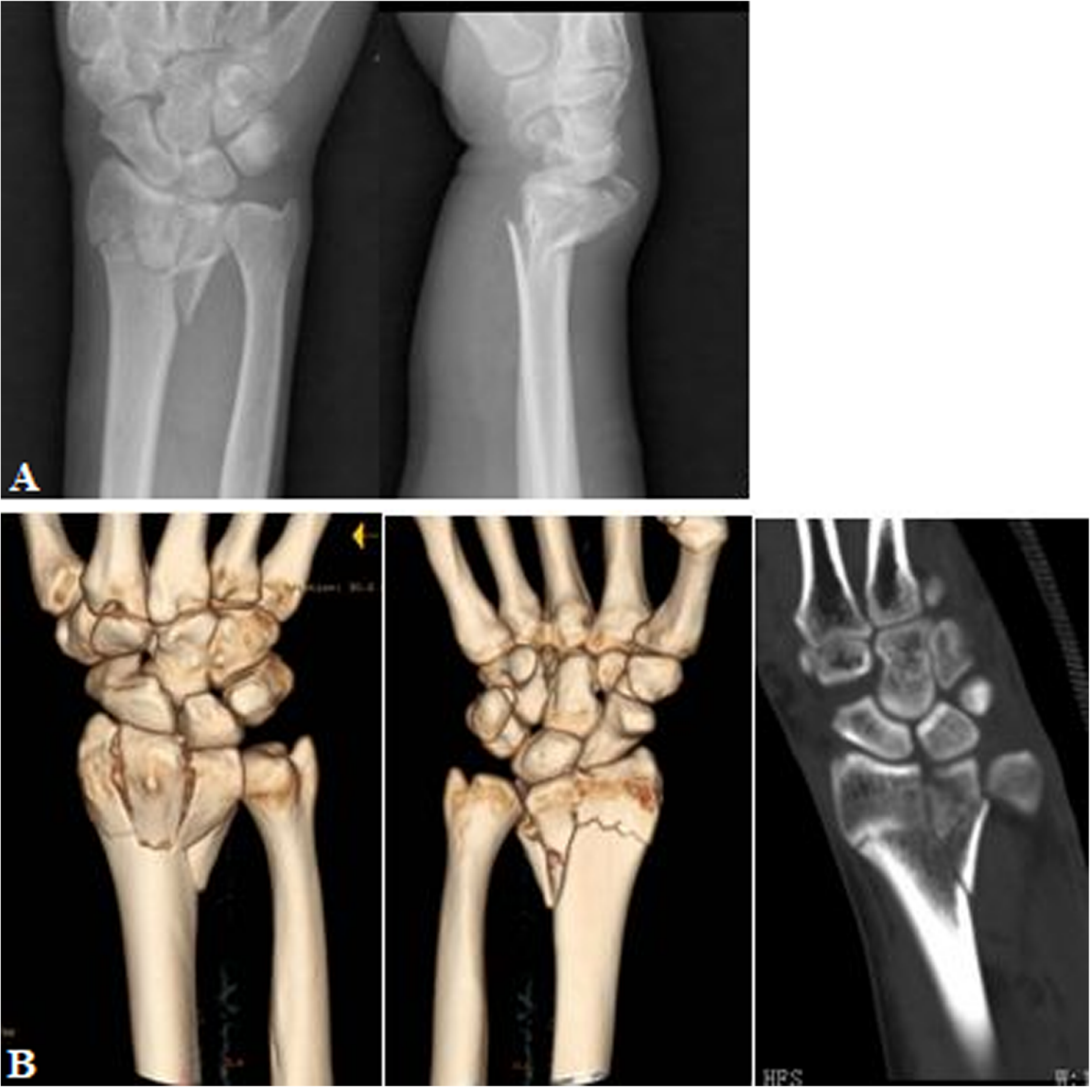
PA X-ray (**a**) and CT scan (**b**) of combined radius column fracture

1. Metaphyseal. The radius column fracture involved the metaphyses of the radius column, specially the bone cortex of the metaphyses presented fracture. No articular surface fracture of the radial column was present (Fig. 8).
2. Articular surface. The radius column fracture involved the articular surface of the radial column. The metaphyses of the radial column presented no fracture (Fig. 9).
3. Combined. The fracture involved both the metaphyses and the articular surface of the radial column (Fig. 10).

### Inter- and intra-observer agreement measurement

Two radiologists were selected and trained with the above classification methods and classified 100 cases of DRDPF patients (40 single column and 60 double or three column) independently. A second classification was conducted 3 months later. When discrepancies occurred between the two observers, the result was determined by a senior doctor. Cohen’s kappa coefficient was then adopted to measure the inter- and intra-observer agreement. The kappa coefficient ranges from − 1 to + 1. Kappa values greater than 0 is characterized as indicating statistical significance, 0.00–0.20 as slight, 0.21–0.40 as fair, 0.41–0.60 as moderate, 0.61–0.80 as substantial, and 0.81–1.00 as almost perfect agreement.

### Statistical analysis

SPSS 16.0 software was used to analyze the data. Comparison between two groups was analyzed with the Chi-square test. A *P* value of < 0.05 was considered significant.

## Results

### Distribution of all the types of fractures

A total of 65 cases were identified as single-column DRDPF, which were all categorized as type B fracture in the AO classification of fractures, among which there were three cases of volar, 13 cases of dorsal, 14 cases of split, and 35 cases of collapse type fractures. There were 406 cases of double-column DRDPF and 89 cases of three-column DRDPF, among which there were 34 cases of type B fractures and 460 cases of type C fractures in the AO classification system. Among the intermediate-column fractures, there were 19 cases of volar, 73 cases of dorsal, 87 cases of split, and 316 cases of collapse type fractures. Among the radius column fracture, there were 130 cases of metaphyseal type which were all classified as type C fracture in the AO classification system; there were 155 cases of articular surface type including 34 cases as type B and 121 cases as type C in the AO classification system; there were 210 cases of combined type which were all classified as type C in the AO classification system. As to the classification of single-, double-, or three-column DRDPF, the kappa coefficient was 0.917–0.937, and the value in the second-time classification was 0.937–0.958. As to the sub-types of volar, dorsal, split, and collapse in the single column, as well as the metaphyseal, articular surface, and combined sub-types in the radius column fracture, the Kappa coefficient was 0.877–0.896 and the value in the second-time classification was 0.916–0.959, showing high agreement. At the 12th month’s follow-up, according to the Gartland–Werley score system for the functionary recovery of the wrist and hand, 519 cases (92.68%) of the patients ranked excellent or good, and 41 cases (7.32%) ranked fair. All the cases were fair results, and the intermediate column of the distal radius was collapse type fractures, showing significant difference between the collapse type and other types (χ2 = 23.460, *P* = 0.000). The excellent and good rate in the single-, double-, and three-column DRDPFs were 93.85%, 92.16%, and 91.01%, respectively (χ2 = 0.018, *P* = 0.991).

## Discussion

The purpose of this study was to investigate the morphological characteristics of different types of DRDPF based on the three-column theory. Previous studies have shown that the three-column theory of the distal radius includes the radial column, ulnar column, and intermediate column. The radial column includes the scaphoid fossa and the radial stem process. The ulnar column is composed of distal ulnar, including the styloid process of the ulna and triangular fibrocartilage complex, and the intermediate column including the lunate fossa and the sigmoid notch [17]. The intermediate column of the distal radius serves primarily for load transmission of the wrist and the axial loads are directed along this column, while the radial column mainly stabilizes the joints and controls the rotation [13, 18]. Since the axial loads are completely or largely transmitted through the lunate to the lunate fossa, articular surface fracture of the lunate fossa or it being the main fracture was commonly seen. Due to the complexity of the axial compression and the difference of the energy of the forces, the position of the wrist, as well as various factors such as the local anatomy and the bone quality of patients, there could be different types of DRDPF with various features [7, 19]. When the radiocarpal joint is in neutral position and the axial loading forces are of low energy, it usually results in articular fracture of the lunate fossa in isolation, which is classified as single-column DRDPF based on the three-column theory. However, it is clinically rare [5, 7]. In more cases, it is common to see a main articular surface fracture of the radius half or and the styloid process of ulna, which is classified as double or three DRDPF in the three-column model. In this study, the incidence of single-column DRDPF and double- or three-column DRDPF in distal radius was 0.7% and 4.0%, respectively. According to the fracture sites and morphological characteristics of single-column DRDPF, it was further divided into four sub-types: volar, dorsal, split, and collapse type fractures [6, 7]. Volar and dorsal single-column DRDPF resulted from the axial loads transmitted to the intermediate column when the wrist was extended or flexed, generally being the avulsion fracture; split fracture, collapse fracture, or both occurred when the carpal joint was in neutral position upon the load transfer. The normal distal radius exhibits a palmer tilt, i.e., the dorsal articular surface is higher than the volar side. The palm usually lands first in falling or stumbling with certain degrees of dorsal carpal extension. Therefore, the dorsal articular surface of the lunate fossa was commonly impacted and therefore is more common than volar type fracture. For example, there were three volar and 13 dorsal type fractures in this study. Split DRDPF was commonly seen in patients with good bone quality and encountered high-velocity stress, mostly younger patients whose articular surface did not easily comminute or collapse, but could result in separation, distal radioulnar joint laxity, and dissociation injuries, while collapse DRDPF usually occurred on patients with poor bone quality, especially elder patients who were more likely to suffer articular comminution and collapse, or younger patients who encountered very high energy axial loading forces, which resulted in articular comminution or collapse and could be associated with longitudinal split fracture to certain degree.

Similarly, based on the characteristics of the fracture sites and the AO classification system, the radius column fracture among the double or three DRDPF was further divided into metaphyseal, articular surface, and combined fractures. The articular type generally resulted from axial loading forces and a certain degree of rotation forces. The rotation forces, which were applied but of low energy, generally leading to mild articular surface fracture of the radial column and being classified as type B or type C fracture in the AO classification system. The metaphyseal type resulted from axial loading forces on the ulnar deviation of the radiocarpal, which not only allowed the forces exert on the intermediate column, but transfer to the radial column, resulting in mild metaphyseal fracture of the radial column. The combined type was caused by a combination of various reasons. The type II fracture in the Melone classification system and type III fracture in the Fernandez classification system regarding distal radial fracture were referred to as die-punch fracture and classified as metaphyseal fracture in this study. Sun [20] and Yamamoto [21] et al. have previously reported articular surface and combined fractures separately. The type II in the Melone classification and type III DRDPF in the Fernandez classification have been widely adopted in literature and books [14, 22].

The patterns and incidence of DRDPF were well explained from the perspectives of mechanisms and anatomical structures in this study method, which is instrumental in understanding the mechanism, fracture sites, morphological changes, and anatomical features of the fractures.

An ideal classification system of fractures should try to include all types of fractures, indicate the fracture characteristics, and be illustrative and intuitive. In this study, we first divided DRDPF into single-, double-, or three-column DRDPF, which included volar, dorsal, split, and collapse type, and the radius column fracture, which were further classified as metaphyseal, articular surface, and combined type. Previously described methods included certain types of DRDPF but were not complete and comprehensive [2–4, 10]. The writer has previously described single-column DRDPF classification, but in this study, the more inclusive classification method of double column was proposed for the first time. This method included all fracture types, indicates the fracture sites and morphological characteristics, and is intuitive, illustrative, and easy to memorize.

Another point for fracture classification is to provide a common, communicative, and consistent language for practitioners. In this study, only a small amount of fractures was not classified in complete agreement by observers. For example, the combined DRDPF was mistaken for metaphyseal type due to the very subtle fractures. Otherwise, the method achieved good agreement between observers with high inter- and intra-coefficient values.

The classification is also meant to provide insight into treatment and prognosis of DRDPF patients. Volar fracture requires volar incision, dorsal fracture requires dorsal incision, and other types require regular volar incision. Split fracture needs depressed fixation by screw and collapse fracture needs percutaneous reduction by leverage and then screws to stabilize [22]. In the AO classification system, single-column DRDPF was classified as B1, B3, or B4 type [19], while metaphyseal and combined fractures of the radius column were referred to as type C and articular surface as type B or C. Based on the principles of the AO classification, type C is more serious than type B, meaning that double or three column has a poorer prognosis and is more serious than single-column DRDPF. The combined and metaphyseal fractures of the radius column have poorer prognosis and were more serious compared with the articular surface type [22]. A typical DRDPF is collapse type of the intermediate column, reminding the surgeons to pay more attention to the reduction and fixation surgery. In the study, the follow-up data also suggested that the different types of fractures had different results. Therefore, this classification method can provide insight into the surgical treatment and prognosis of patients.

## Conclusions

In conclusion, DRDPF is the general term for articular surface fracture of the lunate fossa resulting from axial impact and compressive forces through the lunate bone to the intermediate column. Due to the difference of the nature and energy of the forces, the position of the wrist and the bone quality of patients, the transfer of axial loading forces to the intermediate column of the distal radius could lead to different fracture patterns. Based on the three-column theory of the distal radial fracture proposed by Rikli and Regazzoni, DRDPF could be divided into single-column DRDPF and double- or three-column DRDPFs. The former could be further distinguished as volar, dorsal, split, and collapse types of fracture while the radius column fracture as metaphyseal, articular surface, and combined types of fractures. This classification method can improve the understanding of surgical treatment and prognosis of patients. In the future, we hope to apply this classification method to clinical treatment based on the statistical analysis of clinical results.

## Data Availability

Not applicable.

## Abbreviations

DRDPF: Distal radius die-punch fracture
